# Optimizing Oxylipin Analysis with Liquid Chromatography Mass Spectrometry through Bio-Inert Systems and Ion Funnel Adjustments

**DOI:** 10.64898/2026.07.27.741082

**Published:** 2026-07-28

**Authors:** John T. Shuster, Lingshuang Wu, Jericha Mill, Michaela Morhaus, Nina Yu Fan, Fernando Tobias, Dominique Baldwin, Matthew D. Bruss, Liam Hurley, Michelle Kimple, Adam R. Konopka, Judith Simcox

## Abstract

Oxylipins are potent signaling lipids that affect inflammation, vascular tone, and metabolism, making them relevant in many diseases. Oxylipins are measured with liquid chromatography-mass spectrometry (LC-MS), but challenges in quantification arise due to low abundance and rapid degradation. In this study, we optimize LC-MS methods to improve the quantification of oxylipins in human plasma given growing interest in oxylipins and their impact on clinical research. Plasma samples were obtained from healthy participants and extracted by solid-phase extraction to concentrate the oxylipins. We then utilized a reversed phase targeted LC-MS/MS method using an Agilent 6495D triple quadrupole with transitions for 248 oxylipin species. Ion funnel voltages were set at 50 or 100 volts. Given the rapid degradation of oxylipins with bio-reactive surfaces, we compared both standard and Altura (bio-inert) columns, as well as standard and bio- inert LC setups. We observed that ion funnel parameters significantly alter detectable levels of oxylipins within LC-MS/MS analysis. By decreasing voltages applied to ions inside the ion funnel, signal was increased for most oxylipin species while peak quality was maintained. We also demonstrated that fully bio-inert setups quantify more compounds and show increased levels of some compounds, but fewer epoxyoctadecadienoic acid (EpODE) species. To explore this further, we injected analytical grade alpha-linolenic acid (ALA), the direct precursor of EpODEs, and observed formation of EpODEs within the instrumentation when using stainless steel columns. Our data shows that oxylipins benefit from fully bio-inert systems and optimized pre-mass analyzer parameters. The stainless-steel components of the column may also be contributing to epoxidation reactions of polyunsaturated fatty acids (PUFAs), generating oxylipin species during analysis. Finally, we utilized this method to perform oxylipin analysis in other human tissues including granulocytes, mononuclear cells, erythrocytes, skeletal muscle, and THP-1 cells, a human derived monocyte cell line.

## Full Text Availability

The license terms selected by the author(s) for this preprint version do not permit archiving in PMC. The full text is available from the preprint server.

